# Determination of Highly Sensitive Biological Cell Model Systems to Screen BPA-Related Health Hazards Using Pathway Studio

**DOI:** 10.3390/ijms18091909

**Published:** 2017-09-06

**Authors:** Do-Yeal Ryu, Md Saidur Rahman, Myung-Geol Pang

**Affiliations:** Department of Animal Science and Technology, Chung-Ang University, Anseong, Gyeonggi-do 456-756, Korea; ilky6618@gmail.com (D.-Y.R.); shohagvet@gmail.com (M.S.R.)

**Keywords:** bisphenol-A (BPA) toxicity, cell model types, biomarker, pathway studio, spermatozoa

## Abstract

Bisphenol-A (BPA) is a ubiquitous endocrine-disrupting chemical. Recently, many issues have arisen surrounding the disease pathogenesis of BPA. Therefore, several studies have been conducted to investigate the proteomic biomarkers of BPA that are associated with disease processes. However, studies on identifying highly sensitive biological cell model systems in determining BPA health risk are lacking. Here, we determined suitable cell model systems and potential biomarkers for predicting BPA-mediated disease using the bioinformatics tool Pathway Studio. We compiled known BPA-mediated diseases in humans, which were categorized into five major types. Subsequently, we investigated the differentially expressed proteins following BPA exposure in several cell types, and analyzed the efficacy of altered proteins to investigate their associations with BPA-mediated diseases. Our results demonstrated that colon cancer cells (SW480), mammary gland, and Sertoli cells were highly sensitive biological model systems, because of the efficacy of predicting the majority of BPA-mediated diseases. We selected glucose-6-phosphate dehydrogenase (G6PD), cytochrome b-c1 complex subunit 1 (UQCRC1), and voltage-dependent anion-selective channel protein 2 (VDAC2) as highly sensitive biomarkers to predict BPA-mediated diseases. Furthermore, we summarized proteomic studies in spermatozoa following BPA exposure, which have recently been considered as another suitable cell type for predicting BPA-mediated diseases.

## 1. Introduction

Endocrine disrupting chemicals (EDCs) are exogenous chemicals that can interrupt the endocrine system, from the chemical’s biosynthesis to its elimination, thus producing adverse effects on endocrine homeostasis in the body. Exposure to EDCs is associated with abnormal developmental, neurological, immune, and reproductive functions in both wild animals and humans [1,2]. Among the EDCs, bisphenol-A (BPA) is one of the most ubiquitous and extensively used, which has raised concerns about its prevalence in humans. According to the U.S. Centers for Disease Control and Prevention (CDC), perceptible levels of BPA are detected in urine for over 90% of the population in the U.S. [3]. Recent studies have revealed that BPA is closely associated with several diseases, such as cancer, diabetes, cardiovascular disease, thyroid dysfunction, developmental disorders, miscarriages, and reproductive disorders [4]. Since BPA is regarded as a harmful EDC, which may influence the endocrine system via binding with several physiological receptors preventing the binding of natural hormones, a large number of studies have been conducted, to investigate the effect of BPA and its underlying mechanism for the pathogenesis of diseases using both in vivo and in vitro trials [5,6].

It has been suggested that “omics” disciplines have opened a new avenue for identifying high quality biomarkers, which are reliable for the prediction of various diseases [7]. Most importantly, in the past few years, researchers have successfully engaged in comprehensive proteomic studies of various diseases following exposure to BPA both in vivo and in vitro [8,9,10,11,12]. In addition, bioinformatics has also improved following the development of proteomics, allowing us to make correlations between proteome profiling and interacting proteins, cellular regulation, and associated diseases. Therefore, these comprehensive studies may be considered as accurate and sensitive tools for predicting diseases and hazards to human health. Agarwal et al. (2015) demonstrated that BPA-induced hippocampal neurodegeneration in BPA-treated rats [13]. Moreover, Ge et al. (2014) found 36 proteins were significantly differentially expressed between control and BPA-treated groups in Sertoli cells [12]. Other research groups have also shown that prepubertal exposure to BPA altered mammary gland proteome profiling [14]. A direct comparison of proteome profiling between control and treatment groups may, therefore, be helpful to predict the human health hazards that follow BPA exposure.

BPA has two proposed mechanisms of action: a genomic, and a non-genomic pathway [9,15,16]. In the genomic pathway (nuclear), BPA binds endocrine receptors (ERs) and induces the dimerization of ERs. Subsequently, ER dimers attach to DNA, either directly or indirectly, by binding other transcription factors, including specificity protein 1 and activator protein 1 [15,16]. Alternatively, BPA can affect cell functions through the non-genomic pathway by binding membrane-bound receptors, leading to the activation of kinase signaling pathways [15,17]. During these signaling cascades, the membrane-bound receptor acts with G-protein-coupled receptors (GPCRs), and can cause fast estrogenic signaling by activation of the mitogen-activated protein kinase (MAPK) and phosphatidylinositol 3-kinase (PI3K). Calcium fluctuation, as well as cyclic adenosine monophosphate (cAMP) synthesis [18,19,20,21], may also occur.

Early studies demonstrated that spermatozoa include GPCRs and non-genomic ERs, and thus, spermatozoa have been considered as an alternative model system to investigate the proteome profiling following exposure to BPA, together with the investigation of BPA effects. There is ample experimental data on reproductive disease following exposure to BPA. Higher urinary BPA is significantly correlated with implantation failure, severe endometriosis, low sperm count, increased morphologically abnormal spermatozoa, and DNA damage [22,23,24]. Several studies have examined proteome profiling after BPA exposure, which may also identify precise biomarkers for the prediction of several diseases in this model system.

Therefore, the goal of this study was to identify a more sensitive model system for studying the effects of BPA exposure. Simultaneously, by comparing predicted proteomic biomarkers of BPA exposure in different cell types, we aimed to evaluate more sensitive biomarkers, capable of predicting BPA-mediated disease conditions using a bioinformatics application. In addition, we summarized recently applied proteomics studies in spermatozoa, which could be considered a highly sensitive and effective alternative model for BPA-mediated disease pathogenesis, most importantly, reproductive abnormalities.

## 2. Results and Discussion

### 2.1. Bisphenol-A (BPA) Induces a Wide Range of Diseases in Humans

**Figure 1:**
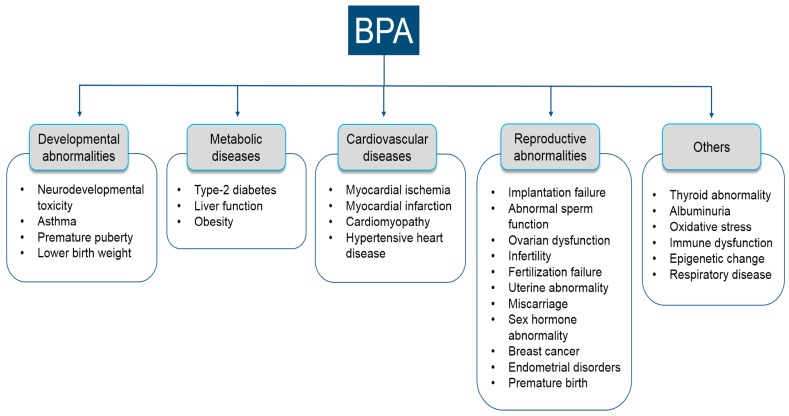
Summary of bisphenol-A-mediated diseases broadly characterized into five major categories.

The effects of BPA on several diseases have been extensively studied, using both animal and human models [4,5,6]. Our search revealed several BPA-mediated diseases, which were broadly characterized into five major categories: developmental, metabolic, reproductive, cardiovascular, and other diseases (Figure 1). In the developmental category, the common diseases included neurodevelopmental toxicity [25,26,27,28,29,30], asthma [31], premature puberty [32,33], and lower birth weight [34,35]. Common diseases in the metabolic category were type-2 diabetes [36,37,38], liver function [39,40], and obesity [41,42,43]. Main diseases in the cardiovascular category included myocardial ischemia [44,45,46], myocardial infarction [39,45], cardiomyopathy [47], and hypertensive heart disease [48]. Major diseases in the reproductive subcategories were implantation failure [23], sperm function [22,49,50], infertility [51,52], ovarian dysfunction [53,54,55], fertilization [56,57], uterine abnormality [58], miscarriages [59,60], abnormal homeostasis of sex hormones [61,62], breast cancer [63,64], endometrial disorders [65,66], and premature birth [65]. In addition, we did not characterize some diseases into specific subcategories, which are also predisposed following BPA exposure, such as thyroid abnormality [67,68], albuminuria [69,70], oxidative stress [71,72], immune dysfunction [73], epigenetic changes [74], and respiratory disease [75]. From these findings, it is clear that exposure to BPA predisposes humans to a wide variety of disease conditions. Several recent studies considered laboratory animal models in vivo, or in vitro cell systems, to help us elucidate BPA-mediated disease conditions. However, more studies should be conducted to better elucidate the underlying molecular mechanisms of such disease pathogenesis. In addition, this study will provide vital information for future development of a theranostic approach to BPA toxicity in clinical conditions.

### 2.2. Compilation of Proteomics Studies Screening BPA Toxicity

Several efforts have been undertaken to evaluate potential adverse health effects of BPA exposure. The recent development of proteomic approaches has provided researchers with useful tools for identifying protein markers of toxic chemical exposure. Proteomics investigations have been conducted in several cell systems to investigate potential biomarkers of BPA exposure, most importantly, in Sertoli cells [12,76], thyroid [77], serum [8], mammary gland [14,78], zebrafish brain [79], rat hippocampus [13], mouse prefrontal cortex [28], colon cancer cells (SW480) [80], and Leydig cells [81].

In this study, we examined the predicted proteomic biomarkers from different cells using the Pathway Studio program. This program is capable of unraveling protein–protein interactions associated with disease biology. In addition, it is capable of providing exclusive statistical application for normalization of data, as well as exact identification of certain genes or proteins in health and diseases. Therefore, it has been considered as a comprehensive biology-based investigational framework, ideal for biomarker analysis and its correspondence relationship with disease modeling.

Our preliminary analysis showed that altered proteins from the majority of the cell types (BPA-exposed) represented an association with BPA-mediated diseases (Tables S1–S9). However, SW480, mammary gland, and Sertoli cells were selected as the most suitable cells for predicting the majority of the diseases caused by BPA (capable of predicting all diseases types; Tables S1–S9). Furthermore, we investigated the altered spermatozoa proteome [9,10] following BPA exposure in detail, which could be another model system for predicting BPA-mediated reproductive diseases.

### 2.3. Efficacy of the BPA-Induced Differential Proteome in SW480 Cells in Predicting BPA-Induced Diseases

**Figure 2:**
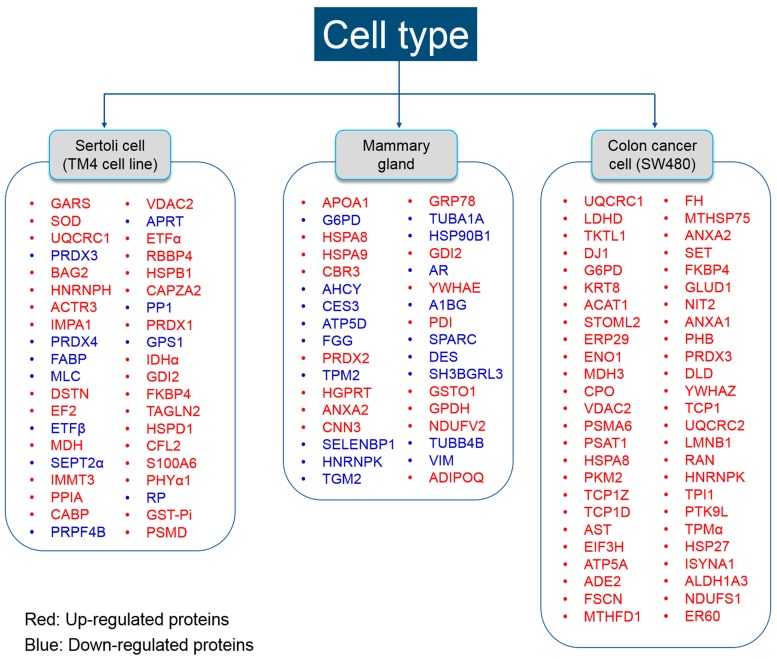
Differentially expressed proteins following bisphenol-A exposure in three cell types. Differentially expressed proteins in the Sertoli cell were summarized according to [12,76]. Differentially expressed proteins in mammary gland cell were summarized according to [14,78]. Differentially expressed proteins in colon cancer cell were summarized according to [80].

The SW480 cell line is derived from human colon cells, and is widely used for studying colon cancer [82,83,84,85]. Chen et al. (2015) showed that exposure to BPA (10^−8^ and 10^−5^ M) for 48 h, in vitro, is capable of promoting metastasis [80], and modifying the protein profile of colorectal cancer. They found 56 differentially expressed proteins following exposure to BPA by MALDI–TOF–MS/MS analysis (see Figure 2).

We analyzed all proteins by the Pathway Studio program, to determine whether a predicted protein marker is capable of detecting BPA-associated diseases significantly. Our data revealed that 9 proteins out of 56 (i.e., MTHFD1, ANXA2, G6PD, ACAT1, UQCRC2, UQCRC1, VDAC2, HNRNPK, and HNRNPL) were significantly associated with major disease categories (developmental, metabolic, reproductive, cardiovascular, and other) (Table 1, *p* < 0.05). Although several other proteins, such as LDLR, PSMA6, ENO1, GLUD1, YWHAZ, SET, and PKM, also represented an association to some BPA-mediated diseases, the relationship was not significant (*p* > 0.05).

Altered MTHFD1 and ANXA2 were significantly associated with reproductive diseases (implantation failure). A study reported by Rozen’s group has also shown that the lack of MTHFDA synthetase significantly affects embryonic defects in female mice. The study demonstrated that disruption of the maternal *MTHFD1* gene damages fetal growth [86]. There are also ample studies that suggest ANXA2 is closely involved with embryo implantation and breast cancer, by acting as an adhesion molecule on the endometrium [87,88,89].

Neurodevelopmental toxicity also can be predicted by G6PD, ACAT1, UQCRC2, UQCRC1, VDAC2, HNRNPK, and HNRNPL proteins, as expressed differentially in the SW480 cell line following BPA exposure. Extensive studies have been conducted to investigate the effects of these proteins and their mechanisms of neurodevelopmental toxicity. Jeng et al. (2013) provided a direct clue that lack of G6PD can cause oxidative DNA damage, which can lead to neurodegenerative disease [90]. ACAT1 is widely known as a critical enzyme, which reversibly converts two units of acetyl-CoA to acetoacetyl-CoA in mitochondria. Therefore, abnormal expression of ACAT1 may affect proper levels of acetyl-CoA, which could cause neurodegeneration [91,92]. On the other hand, alterations of UQCRC1, UQCRC2, and VDAC2 expression, have been reported to be associated with mitochondrial dysfunction, and thus, lead to oxidative stress and reactive oxygen species production [93,94,95]. It is reported that overexpression of UQCRC1 is associated with the development of abnormal behavior, and that *UQCRC2* genes and *VDAC2* are down-regulated in hypertensive rats, and in the hippocampus, under prenatal stress [96,97,98].

These three proteins are also associated with metabolic disease and cardiovascular diseases. VDAC2 is down-regulated in type-2 diabetic mouse liver [99]. Furthermore, VDAC2 has been studied as a regulating factor for cardiac rhythmicity by uptake of mitochondrial Ca^2+^ [100,101]. Nicolas et al. (2017) demonstrated that UQCRC2 is significantly reduced in pancreatic islets of diabetic phenotype mice [102]. In addition, the other studies also have shown that UQCRC2 is differentially expressed in rat insulinoma cell lines, and is significantly reduced under hyperglycemia, which is closely related with cardiac metabolic pathways [103,104]. Therefore, it is tempting to hypothesize that SW480 is a highly sensitive model to screen BPA toxicity, considering its efficacy to predict BPA-mediated disease by differentially expressed protein markers.

### 2.4. Efficacy of BPA-Induced Differential Proteome in Mammary Gland Cells to Predict BPA-Induced Diseases

Betancourt et al. (2012) have reported that prepubertal exposures to BPA-induced altered carcinogenesis in mammary gland cells of rats, which were subsequently predisposed to differential protein expression. They found 18 differentially expressed proteins between BPA-exposed and control rats by 2-dimensional gel electrophoresis followed by MS analysis [14]. In another study, the same research group also investigated differential protein expression in rat mammary glands following exposure to BPA in utero [78]. In the latter study, the differentially expressed proteome was explored by both MALDI-TOF-TOF and LC-MS/MS analysis. Here, we compiled all differentially expressed proteins from both studies. A total of 39 proteins were found to be expressed differentially in rat mammary glands following BPA exposure (see Figure 2). Pathway Studio analysis of these proteins revealed that 9 of 39 proteins (FGG, GTM2, HPRT1, HSPA5, G6PD, ADIPOQ, DES, ANXA2, and HPRT1) represent a significant association with all major disease categories (Table 2, *p* < 0.05).

We found that FGG expression was significantly correlated with reproductive diseases. Lin et al. (2012) have reported that FGG was differentially expressed in the plasma of a uterine leiomyoma patient [105]. Subsequently, FGG was overexpressed in plasma prior to preeclampsia, which is a risky pregnancy syndrome. FGG is widely known as a blood coagulation protein, and thus, might also predict cardiovascular diseases [106].

Three proteins, TGM2, HPRT1, and G6PD, were significantly correlated with neurodevelopmental toxicity. It is reported that TGM2 plays a role in cellular apoptosis, thus, it is involved in various diseases, such as diabetes and neurodegeneration [107,108]. Kang et al. (2011) demonstrated that a lack of HPRT1 is associated with uncontrolled neurodevelopmental pathways [109]. In addition, a study reported by Cristini and colleagues has described that HPRT1 has a critical role in human fetal neurodevelopment [110].

Altered ADIPOQ and HSPA5 expression were significantly correlated with type-2 diabetes. HSPA5 is known to be upregulated in human type-2 diabetes and high-fat-diet-induced diabetes in mice [111,112]. Several researchers have demonstrated that ADIPOQ is closely associated with type-2 diabetes [113,114,115]. Furthermore, although our Pathway Studio program did not show a significant association, these two proteins are also closely linked with cardiovascular diseases. ADIPOQ has been studied as a vital factor in the cardiac metabolic pathway that may be helpful in treating diabetes and cardiovascular disease [116,117]. It is reported that HSPA5 is significantly increased in the hypertensive heart, which may lead to heart failure [118,119]. Altered FGG is found in men with myocardial infarction, arterial disease, and heart failure through blood coagulation [120,121]. DES is also considered as a potential biomarker for the prediction of cardiovascular diseases. It is reported that aberrant DES aggregation can lead to cardiac diseases [122].

### 2.5. Efficacy of BPA-Induced Differential Proteome in Sertoli Cell to Predict BPA-Induced Diseases

Sertoli cells are the “nurse” cells of the testis that help in spermatogenesis and regulate uninterrupted production of spermatozoa. Because of their important role in spermatogenesis, Sertoli cells are the foremost target of toxicant-induced reproductive abnormalities. Ge et al. (2014) have analyzed the effects of BPA on Sertoli cell (TM4 cell line) proliferation and altered proteome by MALDI-TOF-MS/MS analysis [12]. Others have also described the altered proteome in Sertoli cells (TM4 cell line) treated with BPA by MALDI-TOF-MS/MS analysis [76]. Here, we collected all differentially expressed proteins from both studies (Figure 2). A total of 42 differentially expressed proteins were analyzed by the Pathway Studio program to investigate the interactions between proteins and diseases. Five different proteins (e. g., HSPB1, SOD1, UQCRC1, VDAC2, and HSPB1) were found to be associated with various BPA-associated diseases such as reproductive, developmental, metabolic, and cardiovascular, following exposure to BPA (Table 3, *p* < 0.05).

Our Pathway Studio results revealed that HSPB1 is significantly associated with reproductive disease (implantation failure). It is reported that HSPB1 protein is overexpressed in uterine distension during pregnancy, as well as inducing the distension of the myometrium, owing to the growing fetus [123,124].

In the Sertoli cell system, we also found that type-2 diabetes and cardiovascular diseases could be predicted by three different proteins (UQCRC1, VDAC2, and SOD1). Neves et al. (2012) described the vital role of SOD1 in preventing cardiovascular diseases by countering to oxidative stress caused by the type-2 diabetes [125]. Consistent with this finding, other research groups have demonstrated that the distribution of SOD1 is different between diabetes patients and healthy controls [126]. Moreover, it is reported that SOD1 deficiency has critical implications for the pathogenesis of cardiac diseases [127]. Therefore, considering the efficacy of the three cell types (SW480, mammary gland, and Sertoli cells) in predicting BPA-exposure-related disease, it is tempting to conclude that all cells provide considerable efficacy to predict BPA toxicity. Similar studies should be conducted, considering both in vitro and in vivo experimental design, to increase the specificity and efficacy of the identified biomarkers to predict BPA-associated diseases.

### 2.6. Commonly Expressed Proteins in Three Major Cell Types Following BPA Exposure

Among the various protein biomarkers, we noticed that G6PD, UQCRC1, and VDAC2 were associated with the same disease categories in more than one of the three cell types. G6PD is associated with neurodevelopmental diseases in both mammary gland and SW480 cell lines. UQCRC1 and VDAC2 were associated with neurodevelopmental disease, type-2 diabetes, and cardiovascular disease in both Sertoli cell and SW 480 cell lines (Table 4).

A large number of studies have been conducted to investigate the effect of G6PD and its underlying mechanisms. It is reported that G6PD is a catalytic enzyme in the pentose phosphate pathway to provide the energy to cells by maintaining NADPH [128]. G6PD deficiency is a common disease worldwide known commonly as favism, which can lead to hemolysis and other conditions, such as hemolytic anemia, diabetes, and neonatal jaundice [129]. It is worth noting that G6PD deficiency is caused by an X-chromosome abnormality, which may be damaged by BPA exposure. Many studies have reported that BPA has an impact on G6PD [129,130,131,132]. As aforementioned, UQCRC1 and VDAC2 play a vital role in the mitochondrial respiratory chain, resulting in decreased ATP synthesis, increased oxidative stress, and reactive oxygen species production [93,94,95]. BPA is reported to be significantly related to the generation of ATP, oxidative stress, and especially mitochondrial dysfunction, in a manner similar to the biological functions of these proteins [9,10,133,134,135,136]. Based on these previous studies, we can anticipate biological functions or associated diseases of specific proteins. G6PD, UQCRC1, and VDAC2 are well-studied proteins in both signaling pathways and diseases; therefore, these three proteins could become highly utilized biomarkers to predict diseases that are closely associated with BPA exposure. However, further studies are required to confirm the safety and efficacy of these biomarkers for possible clinical applications. Interestingly, we did not find any single protein common in all cell types. This may be because the exposure scheme of BPA, and the doses of exposure in each study, were different.

### 2.7. Efficacy of BPA-Induced Differential Proteome in Spermatozoa to Predict BPA-Induced Diseases

An intensive search in literature revealed that mature spermatozoa contain both GPCRs and non-genomic ERs; thus, this is an excellent model to screen the molecular mechanism of EDC action [130,137,138]. Although there are numerous studies where spermatozoa were used to investigate the action of EDCs including BPA, few studies have been conducted to demonstrate the proteomic change of spermatozoa following BPA exposure. Recently, we introduced spermatozoa as a model cell to investigate protein biomarkers following both in vitro and in vivo exposure to BPA. This model is highly sensitive because mature mammalian spermatozoa are mostly incapable of protein synthesis; thus, predicted protein biomarkers in spermatozoa provide considerable constancy for use in clinical conditions [139,140,141].

**Figure 3:**
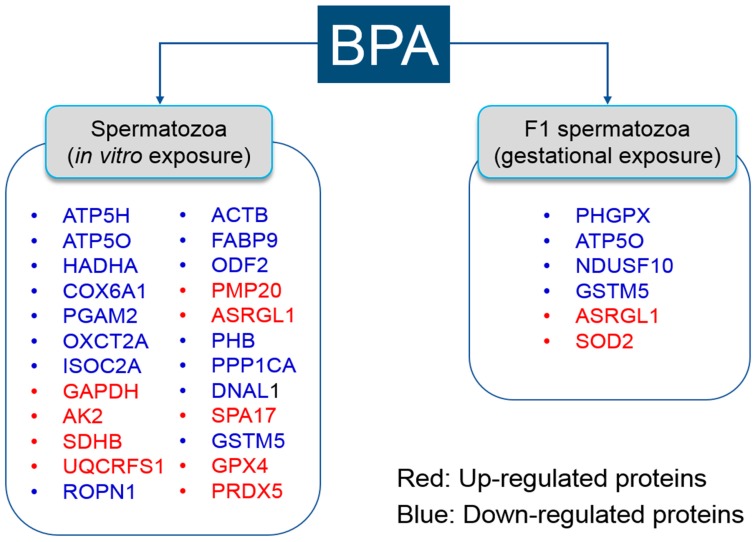
List of differentially expressed proteins following bisphenol-A exposure in spermatozoa. Differentially expressed proteins in the spermatozoa in vitro were summarized according to [10,135]. Differentially expressed proteins in F1 spermatozoa following gestational exposure to bisphenol-A were summarized according to [9].

Our research group demonstrated that BPA affects sperm function, fertility, and proteome profiles in mice in vitro [10,135]. Subsequently, we have also reported that sperm function, fertility, and proteome were influenced in F1 spermatozoa following gestational exposure to BPA [9]. Here, we compiled all differentially expressed proteins from both in vitro and in vivo studies (Figure 3).

We then analyzed them to investigate the linkage between altered protein profiles and BPA-mediated diseases by the Pathway Studio program. Three altered proteins (e.g., UQCRFS1, SDHB, and ATP5O) out of 30 were matched with BPA-mediated diseases, as shown in Table 5.

UQCRFS1, SDHB, and ATP5O were significantly associated with both neurodevelopmental toxicity and type-2 diabetes (*p* < 0.05). In addition, both UQCRFS1 and ATP5O presented a statistically significant association with cardiovascular diseases (*p* < 0.05). However, contrary to our expectation, there was no significant association between the differentially expressed proteins and BPA-mediated reproductive diseases. The statistical value output, given by Pathway Studio, is compiled based on a curated pathway, which is derived from millions of full-text articles, 25 million abstracts, and more than 164,000 clinical trials. Therefore, this may be because the exact pathways of specific reproductive diseases are not yet well established compared to other diseases. Despite many studies already reporting the association between specific proteins and fertility, relatively few studies are performed on the underlying mechanism of specific reproductive diseases. For instance, several studies have extensively reported that PRDXs plays a vital role in fertility through preventing oxidative stress; however, the underlying mechanisms are not fully understood [142,143,144,145]. Therefore, we decided to link the differentially expressed proteins to BPA-mediated reproductive diseases manually, using the Pathway Studio program without statistical analysis.

**Figure 4:**
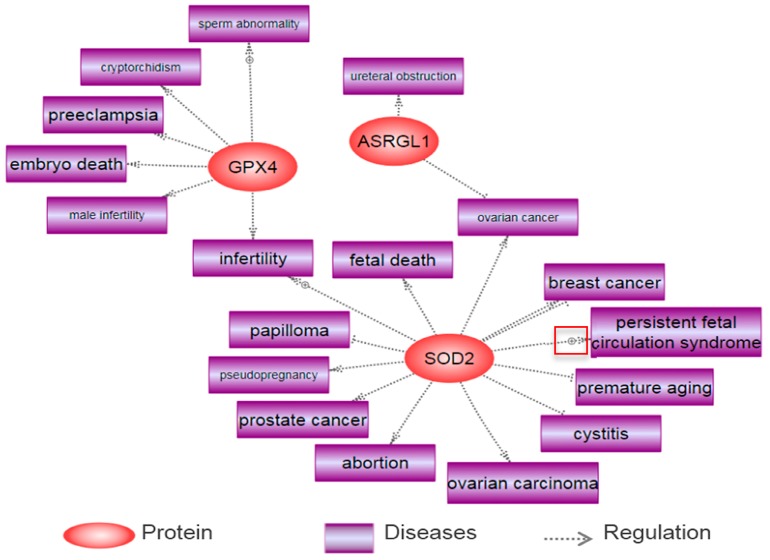
Reproductive diseases associated with several proteins following in vitro exposure to bisphenol-A in spermatozoa.

We found three proteins (GPX4, ASRGL1, and SOD2) out of six differentially expressed proteins in F1 spermatozoa following BPA exposure were related to several reproductive diseases using the Pathway Studio program. The major diseases include sperm abnormality, cryptorchidism, preeclampsia, male infertility, embryo death, ureteral obstruction, ovarian cancer, cystitis, breast cancer, prostate cancer, abortion, pseudo pregnancy, ovarian carcinoma, papilloma, fetal death, and infertility. These have been summarized in Figure 4.

Imai et al. (2009) reported that abnormalities of spermatozoa and seminiferous tubules are found in spermatocyte-specific GPX4 knockout mice [146]. Consistently, it is reported that the deficiency of GPX4 in these mice resulted in embryonic death through oxidative stress, which may cause male infertility [147,148]. SOD2 is a widely known effective antioxidant enzyme, which can prevent oxidative stress. Several studies have revealed that this protein has an impact on reproductive diseases. Uriu-Adams et al. (2005) reported that disruption of SOD leads to fetal death in mice, and other research also revealed that a genetic polymorphism in SOD determines the risk of infertility in patients [149,150]. In addition, it is reported that ASRGL causes ureteral obstruction through inflammatory complication of acute pancreatitis [151]. Few studies have been conducted to demonstrate the underlying mechanisms of action of specific proteins on reproductive diseases, although the relationship between these proteins and disease was insignificant.

**Figure 5:**
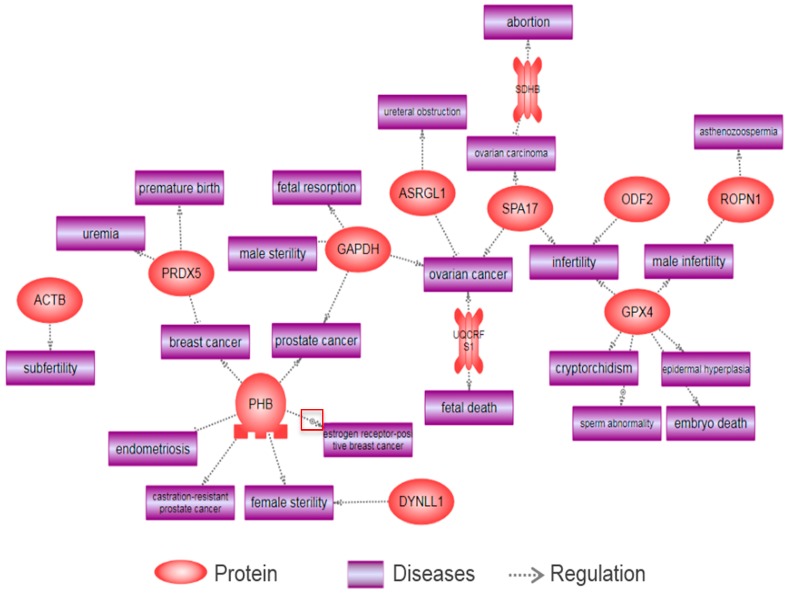
Reproductive diseases associated with several proteins following gestational exposure to bisphenol-A in F1 spermatozoa.

Using an in vitro approach, we found that BPA is capable of altering a total of 24 proteins in spermatozoa. Subsequently, 13 differentially expressed proteins (e.g., ACTB, DYNLL1, ASRGL1, PHB, SPA17, ATP5O, GAPDH, PRDX5, GPX4, UQCRFS1, ROPN1, ODF2, and SDHB) were associated with various reproductive diseases, such as ureteral obstruction, endometriosis, subfertility, prostate cancer, breast cancer, ovarian cancer, male sterility, fetal resorption, abortion, ovarian carcinoma, female sterility, infertility, asthenozoospermia, fetal death, male infertility, sperm abnormality, cryptorchidism, embryo death, uremia, and premature birth, as shown by Pathway Studio (Figure 5).

Consistent with the differentially expressed proteins of F1 spermatozoa, both GPX4 and ASRGL1 were altered by BPA exposure. SPA17 has been reported to induce tissue-specific malignancy in human epithelial ovarian cells [152]. Furthermore, GAPDH, an important catalytic enzyme for glycolysis, has been studied in relation to reproductive diseases. Riley has described that inhibition of GAPDH affects fetal resorption [153]. In another study, the same research group reported that inactive GAPDH causes the apoptosis of blastocysts, and thus results in fetal resorption [154]. Furthermore, it is reported that GAPDH has impacts on male sterility, prostate cancer, and ovarian cancer [155,156,157]. PHB is also associated with several reproductive diseases. He et al. (2015) showed that modification of the *PHB* gene caused abnormal development and estrogen insensitivity in mice uteri, and resulted in sterility [158]. In addition, another study demonstrated that PHB plays a vital role in the initiation of prostate cancer and early androgen-independent tumors [159]. It is reported that ROPN1 deficiency affects sperm motility by PKA-dependent signaling processes, resulting in male infertility [160]. DYNLL1 has been reported to have an impact on female sterility [161]. Subsequently, it has been reported that PRDX5 has a positive effect on breast cancer by regulating oxidative stress [162]. León et al. (2007) described that SDHB is downregulated in pollen abortions in Arabidopsis [163]. Moreover, other proteins (ODF2, UQCRFS1, and ACTB) are involved in infertility, ovarian cancer, fetal death, and subfertility [164,165,166,167].

In summary, although we could not demonstrate a statistical association between BPA-mediated reproductive diseases and altered protein expression following BPA exposure in spermatozoa, we found that several differentially expressed proteins are correlated with reproductive diseases, according to our bioinformatic data. Therefore, we speculate that spermatozoa could be considered as a potential biological cell model system to predict BPA-mediated reproductive diseases more specifically. However, further studies should be conducted for screening the alteration of the proteome within reproductive diseases, and discovering the underlying mechanisms and pathways of various reproductive diseases. On the other hand, the majority of the cells revealed a sex-based difference following exposure to chemicals and microbial stressors [168]. These differences may affect BPA-induced proteomic profiles in male and female cells in a sex-specific manner. Therefore, rigorous preclinical study with the focus on sex or gender must be considered to develop more specific guidelines of a chemical toxicity [169].

## 3. Materials and Methods 

### 3.1. Identification of BPA-Mediated Diseases

The PubMed search engine was used to thoroughly search the MEDLINE database for literature to identify BPA-mediated disease/disease conditions. Briefly, we compiled published studies that were conducted as epidemiological studies on human health hazards mediated via either direct or indirect BPA exposure. The various diseases and disorders identified were categorized into five major categories: developmental abnormalities, metabolic disease, cardiovascular disease, reproductive diseases, and others.

### 3.2. Identification of Model Cell Systems to Identify Proteomic Biomarkers of BPA Exposure

The PubMed search engine was also used to thoroughly identify scientific literature regarding proteomic studies on several cell types, to investigate the differentially expressed proteins following exposure to BPA both in vitro and in vivo.

### 3.3. Analysis of Disease Pathways by Differentially Expressed Proteins

The differentially expressed proteins following BPA exposure in each cell type were analyzed by the computation bioinformatics program Pathway Studio^®^ 9.0 (Ariadne Genomics, Rockville, MD, USA), to demonstrate whether the proteomics biomarker in different cells could predict BPA-mediated diseases/disease conditions. Briefly, protein names (symbols) were entered into the program to determine significantly matching diseases for each differentially expressed protein, based on the information extracted from the NCBI PubMed database. Related diseases associated with each differentially expressed protein were re-confirmed using a PubMed Medline hyperlink that was embedded in each node. Fisher’s exact test was used to determine whether a disease was statistically correlated with the target protein. *p* < 0.05 was considered statistically significant.

## 4. Conclusions

Comprehensive proteomics approach has already been developed as a high-throughput technique, which is helpful to analyze numerous altered proteins in pathological and toxicology studies. A direct comparison of the differentially expressed proteome following exposure to toxic agents/BPA could help identify potential protein biomarkers that predict the abnormality from exposure to specific EDCs. Simultaneously, these protein biomarkers can be linked to extensive biological functions, interacting proteins, and diseases based on these “footprints”. Here, we showed that SW480, mammary gland, and Sertoli cells are the most suitable cell system models to predict BPA-mediated diseases among other examined cell types, such as thyroid, serum, zebrafish brain, rat hippocampus, prefrontal cortex of mice, and Leydig cells. Subsequently, we also demonstrated that G6PD, UQCRC1, and VDAC2 may be the most sensitive and highly utilized protein biomarkers, which can predict all major disease categories following BPA exposure. Furthermore, we showed that various differentially expressed proteins in spermatozoa following BPA exposure are also closely related with many disease categories, including reproductive diseases. Therefore, it seems that spermatozoa might be a good potential biological cell model type to predict BPA-mediated reproductive diseases. However, further studies are needed to evaluate and enhance the specificity and efficacy of potential biomarkers to predict BPA-mediated diseases, and to establish the pathways and mechanisms related to reproductive diseases.

## Figures and Tables

**Table 1:** Summary of bisphenol-A-induced differential proteomes in human cancer colon cell (SW480) and their association with bisphenol-A-mediated diseases. “-” = No specific search results were found.

Major Disease Category	Specific Disease	Overlapping Entitles	*p*-Value
Reproductive	Implantation failure	MTHFD1, ANXA2	<0.01
Developmental	Neurodevelopmental toxicity	G6PD, ACAT1, UQCRC2, UQCRC1, VDAC2, HNRNPK, HNRNPL	<0.01
Metabolic	Type-2 diabetes	UQCRC2, UQCRC1, VDAC2	<0.01
Cardiovascular	-	UQCRC2, UQCRC1, VDAC2, MTHFD1, ANXA2	<0.01

**Table 2:** Summary of bisphenol-A-induced differential proteome in mammary gland cells and their association with bisphenol-A-mediated diseases.

Major Disease Category	Specific Disease	Overlapping Entitles	*p*-Value
Reproductive	Implantation failure	FGG	<0.05
Developmental	Neurodevelopmental toxicity	TGM2, HPRT1, G6PD	<0.05
Metabolic	Type-2 diabetes	ADIPOQ, HSPA5	<0.05
Cardiovascular	Cardiomyopathy	FGG, DES, ANXA2	<0.05

**Table 3:** Summary of bisphenol-A-induced differential proteome in Sertoli cells (mouse TM4 cells) and association with bisphenol-A-mediated diseases.

Major Disease Category	Specific Disease	Overlapping Entities	*p*-Value
Reproductive	Implantation failure	HSPB1	<0.05
Developmental	Neurodevelopmental toxicity	SOD1, UQCRC1, VDAC2	<0.05
Metabolic	Type-2 diabetes	UQCRC1, VDAC2, SOD1	<0.05
Cardiovascular	Myocardial ischemia	UQCRC1, VDAC2, SOD1	<0.05

**Table 4:** Commonly expressed proteins in mammary gland, Sertoli, and human SW480 cells following exposure to BPA and their efficacy to predict BPA-mediated diseases. “-” = No specific search results were found.

Major Disease Category	Specific Disease	Cell Type			*p*-Value
		Mammary Gland	Sertoli Cell	SW480	
Reproductive	-	-	-	-	-
Developmental	Neurodevelopmental disease	G6PD	UQCRC1, VDAC2	G6PD, UQCRC1, VDAC2	<0.05
Metabolic	Type-2 diabetes	-	UQCRC1, VDAC2	UQCRC1, VDAC2	<0.05
Cardiovascular disease	Myocardial ischemia	-	UQCRC1, VDAC2	UQCRC1, VDAC2	<0.05

**Table 5:** Summary of bisphenol-A-induced differential proteomes in spermatozoa and their association with bisphenol-A-mediated diseases. “-” = No specific search results were found.

Major Disease Category	Specific Disease	Overlapping Entities	*p*-Value
Reproductive	-	-	-
Developmental	Neurodevelopmental toxicity	UQCRFS1, SDHB, ATP5O	<0.01
Metabolic	Type-2 diabetes	UQCRFS1, SDHB, ATP5O	<0.01
Cardiovascular	Myocardial ischemia	UQCRFS1, ATP5O	<0.01

## Supplementary Materials

Supplementary materials can be found at www.mdpi.com/1422-0067/18/9/1909/s1.

## Abbreviations

BPA	Bisphenol-A
CDC	Centers for Disease Control
EDC	Endocrine Disrupting Chemical
ER	Endocrine Receptor
GPCR	G-protein-Coupled Receptors
MAPK	Mitogen-Activated Protein Kinase
PI3K	Phosphatidylinositol 3-Kinase
