# Supplementary material for: Determination of Highly Sensitive Biological Cell Model Systems to Screen BPA-Related Health Hazards Using Pathway Studio
Source: Int J Mol Sci. 2017 Sep 6;18(9):1909. doi: 10.3390/ijms18091909 (PMC5618558; doi:10.3390/ijms18091909)
Supplement: Supplementary file 1 [file ijms-18-01909-s001.pdf]

Supplementary materials: Determination of Highly Sensitive Biological Cell Model Systems to Screen BPA-Related Health Hazards using Pathway Studio

Do-Yeal Ryu, Md Saidur Rahman and Myung-Geol Pang

Table S1. Summary of BPA-induced differential proteome in human SW480 cells and their association with BPA-mediated diseases.

Major disease category	Specific disease	Overlapping entitles	*p*-Value
Reproductive	Implantation failure	MTHFD1, ANXA2	< 0.01
Developmental	Neurodevelopmental toxicity	G6PD, ACAT1, UQCRC2, UQCRC1, VDAC2, HNRNPK, HNRNPL	< 0.01
Metabolic	Type-2 diabetes	UQCRC2, UQCRC1, VDAC2	< 0.01
Cardiovascular	-	UQCRC2, UQCRC1, VDAC2, MTHFD1, ANXA2	< 0.01

Table S2. Summary of BPA-induced differential proteome in mammary gland cells and their association with BPA-mediated diseases.

Major disease category	Specific disease	Overlapping entitles	*p*-Value
Reproductive	Implantation failure	FGG	< 0.05
Developmental	Neurodevelopmental toxicity	TGM2, HPRT1, G6PD	< 0.05
Metabolic	Type-2 diabetes	ADIPOQ, HSPA5	< 0.05
Cardiovascular	Cardiomyopathy	FGG, DES, ANXA2	< 0.05

Table S3. Summary of BPA induced differential proteome in Sertoli cells (mouse TM4 cells) and association with BPA-mediated diseases.

**Major disease category**	**Specific disease**	**Overlapping entitles**	***P*-value**
Reproductive	Implantation failure	HSPB1	< 0.05
Developmental	Neurodevelopmental toxicity	SOD1, UQCRC1, VDAC2	< 0.05
Metabolic	Type-2 diabetes	UQCRC1, VDAC2, SOD1	< 0.05
Cardiovascular	Myocardial ischemia	UQCRC1, VDAC2, SOD1	< 0.05

Table S4. Summary of BPA induced differential proteome in hippocampus of the rat brain and association with BPA-mediated diseases.

**Major Disease Category**	**Specific Disease**	**Overlapping entitles**	***p*-Value**
Reproductive	-	-	-
Developmental	Neurodevelopmental toxicity	GDA, PKM, TUBB2A, TUBA1B, IL9, PEBP1	< 0.05
Metabolic	Type-2 diabetes	-	-
Cardiovascular	-	DDAH1	< 0.05

Table S5. Summary of BPA induced differential proteome in mouse Leydig cell line (TM3) and association with BPA-mediated diseases.

**Major Disease Category**	**Specific Disease**	**Overlapping entitles**	***p*-Value**
Reproductive	-	-	-
Developmental	Neurodevelopmental toxicity	COL1A1, CFL1, VCL	< 0.01
Metabolic	Type-2 diabetes	AKR1B1	< 0.05
Cardiovascular	-	-	-

Table S6. Summary of BPA induced differential proteome in mouse prefrontal cortex and association with BPA-mediated diseases.

Major Disease Category	Specific Disease	Overlapping entitles	*p*-Value
Reproductive	-	-	-
Developmental	Neurodevelopmental toxicity	ATP6V1B1	< 0.05
Metabolic	Type-2 diabetes	ATP6V1B1	< 0.05
Cardiovascular	Vasospasm	ATP6V1B1	< 0.05

Table S7. Summary of BPA induced differential proteome in rat serum and association with BPA-mediated diseases.

Major Disease Category	Specific Disease	Overlapping entitles	*p*-Value
Reproductive	-	-	-
Developmental	Neurodevelopmental toxicity	LBR, SMC3	< 0.05
Metabolic	Type-2 diabetes	ITGA6, DLK1	< 0.05
Cardiovascular	-	-	-

Table S8. Summary of BPA induced differential proteome in zebrafish brain and association with BPA-mediated diseases.

Major Disease Category	Specific Disease	Overlapping entitles	*p*-Value
Reproductive	-	-	-
Developmental	Neurodevelopmental toxicity	ATP2A2, SLC25A4, IARS	< 0.05
Metabolic	Type-2 diabetes	ACTN2, ATP2A2, SLC25A4	< 0.01
Cardiovascular	Cardiomyopathy	ACTN2, ATP2A2, SLC25A4	< 0.01

Table S9. Summary of BPA induced differential proteome in thyroid of female mice and association with BPA-mediated diseases.

**Major Disease Category**	**Specific Disease**	**Overlapping entitles**	***p*-Value**
Reproductive	-	-	-
Developmental	Neurodevelopmental toxicity	VCP, HSPA5	< 0.01
Metabolic	Type-2 diabetes	HSPA5	< 0.05
Cardiovascular	-	-	-
